# Expression of PD-L1 and PD-L2 and Their Association with IFN-γ/STAT1/STAT3 Signaling in Human Clear Cell Renal Cell Carcinoma (ccRCC)

**DOI:** 10.3390/jcm15114384

**Published:** 2026-06-05

**Authors:** Gábor Kónya, Ali Shammas, Erzsébet Szabó, Róbert Tupicza, Seyed Behrad Piran, Krisztián Szegedi, Anna Vass, Éva Juhász, József Király, Balázs Dezső, Mahua Choudhury, Zsuzsanna Szabó, Gábor Halmos

**Affiliations:** 1 Department of Biopharmacy, Faculty of Pharmacy, University of Debrecen, 4002 Debrecen, Hungary; konya.gabor@pharm.unideb.hu (G.K.); shammas.ali@pharm.unideb.hu (A.S.); behradpiran99@gmail.com (S.B.P.); vass.anna@pharm.unideb.hu (A.V.); kiraly.jozsef@pharm.unideb.hu (J.K.); 2 Doctoral School of Pharmaceutical Sciences, University of Debrecen, 4032 Debrecen, Hungary; 3 Department of Pharmacology, Faculty of Pharmacy, University of Debrecen, 4002 Debrecen, Hungary; erzsebet.szabo@med.unideb.hu (E.S.); tupicza.robert@pharm.unideb.hu (R.T.); 4 HUN-REN-DE Pharmamodul Research Group, University of Debrecen, 4032 Debrecen, Hungary; 5 Department of Urology, Faculty of Medicine, University of Debrecen, 4032 Debrecen, Hungary; szegedi.krisztian@med.unideb.hu; 6 Department of Pediatrics, Faculty of Medicine, University of Debrecen, 4032 Debrecen, Hungary; juhasze@med.unideb.hu; 7 Department of Pathology, Faculty of Medicine, University of Debrecen, 4032 Debrecen, Hungary; bdezso@med.unideb.hu; 8 Department of Pharmaceutical Sciences, Irma Lerma Rangel School of Pharmacy, Texas A&M Health Science Center, College Station, TX 77845, USA; mchoudhury@tamu.edu

**Keywords:** ccRCC, PD-1, PD-L1, PD-L2, TME, STAT1, STAT3, IFN-γ signaling, immune checkpoint

## Abstract

**Background:** Programmed cell death protein-1 (PD-1) and its ligands, PD-L1 and PD-L2, constitute a central immune checkpoint pathway that regulates T-cell activity and tumor immune escape, while their relationship with STAT signaling remains incompletely understood in ccRCC. **Methods:** We analyzed 27 paired ccRCC and adjacent non-tumorous human kidney tissue samples. mRNA levels of PD-1, PD-L1, PD-L2, STAT1, and STAT3 were quantified by RT-qPCR. In addition, representative human ccRCC cell lines (CAKI-2 and A-498) were treated with IFN-γ to assess the time-dependent modulation of immune checkpoint molecules and STAT pathway activation. **Results:** PD-L1 and PD-L2 were significantly upregulated in tumor tissues compared with adjacent normal kidney tissue. Exploratory observation suggests grade dependent increase. Whereas PD-1 was predominantly downregulated, IFN-γ treatment induced a rapid transcriptional upregulation of PD-L1 and PD-L2 in RCC cell lines, with maximal protein accumulation observed at 72 h. STAT1, but not STAT3, exhibited dynamic induction following IFN-γ stimulation, showing temporal association with PD-L1 and PD-L2 upregulation, indicating cell-line-specific regulatory effects. Correlation analyses confirmed a strong correlation between PD-1 and its ligands, whereas STAT1 and STAT3 expression showed no direct association with PD-L1 or PD-L2 levels in cancer samples. **Conclusions:** Our findings demonstrate that PD-L1 and PD-L2 are frequently upregulated in ccRCC and dynamically regulated by IFN-γ/STAT1-dependent signaling. Our results provide additional insight into the mechanisms of immune escape and underscore the potential of integrated profiling of PD-1 ligands and STAT signaling to guide personalized immunotherapeutic strategies in ccRCC.

## 1. Introduction

Renal cell carcinoma (RCC) is the most common malignancy of the kidney, accounting for approximately 2–3% of all adult cancers worldwide. Clear cell RCC (ccRCC) represents the predominant histological subtype and is characterized by pronounced immune cell infiltration and a highly immunomodulatory tumor microenvironment (TME) [1]. Despite improvements in surgical techniques and the development of targeted therapies, advanced and metastatic RCCs remain largely incurable [2].

Over the past decade, immune checkpoint inhibitors (ICIs) targeting the programmed cell death-1 (PD-1) pathway have fundamentally transformed the treatment of RCC, leading to durable clinical responses in a subset of patients [3,4,5,6]. Immune evasion in RCC involves multiple components of the TME, including tumor-associated macrophages, regulatory T cells, and cancer-associated fibroblasts, as well as key immune checkpoints such as PD-1/PD-L1 and Cytotoxic T-Lymphocyte-Associated Protein 4 (CTLA-4) [7]. Clinical trials have established ICIs as the standard of care, either as monotherapy or in combination with tyrosine kinase inhibitors or mTOR inhibitors, demonstrating improved response rates and patient survival [7]. Nevertheless, primary and acquired resistance remain major challenges, often driven by adaptive signaling pathways and immune exhaustion. Emerging strategies, including dual checkpoint blockade, combination therapies, and biomarker-guided approaches, are being explored to overcome resistance and optimize patient outcomes [7,8].

The PD-1 immune checkpoint axis comprises the inhibitory receptor PD-1, expressed predominantly on activated T lymphocytes, and its ligands PD-L1 (CD274) and PD-L2 (PDCD1LG2). Engagement of PD-1 by either ligand attenuates T-cell receptor signaling, reduces cytokine production, and impairs cytotoxic activity, ultimately promoting T-cell exhaustion and immune tolerance [3,9,10]. Tumors frequently exploit this pathway by upregulating PD-1 ligands, suppressing antitumor immune responses, and facilitating disease progression [3,9,10].

PD-L1 has been the most extensively investigated ligand in RCC. Elevated PD-L1 expression correlates with aggressive tumor features, increased metastatic potential, and poor prognosis [11]. However, static PD-L1 assessment alone may not reliably predict response to PD-1/PD-L1 blockade, as certain responses are observed in both PD-L1 positive and PD-L1 negative tumors. PD-L2, expressed by tumoral, stromal, and endothelial cells, often correlates with PD-L1 but can be present independently [12]. Clinical studies indicate that combined PD-L1/PD-L2 positivity predicts higher response rates and improved progression-free and overall survival, emphasizing the value of evaluating both ligands for biomarker-driven patient stratification [12]. Nevertheless, systematic analyses of PD-L2 in paired human RCC tissues remain limited, and its potential independent role in immune suppression is still poorly defined.

Cancer immunotherapy exploits the patient’s immune system to target tumor cells, but the immunosuppressive TME significantly shapes therapeutic efficacy [13]. Genomic instability in RCC can generate somatic mutations, some of which produce neoantigens recognized by T cells, linking mutational load to immunotherapy response [14]. Cytokine-rich TMEs with infiltrating T cells, macrophages, and dendritic cells induce PD-L1 and PD-L2 expression in tumoral and stromal cells, as seen in non-small cell lung cancer (NSCLC) and melanoma [12]. This dynamic regulation is influenced by tumor type, TME composition, and cytokine milieu [15].

Expression of PD-L1 and PD-L2 is regulated by genetic, epigenetic, and transcriptional mechanisms. Cytokines, particularly interferon-γ (IFN-γ), induce ligand expression via JAK–STAT signaling, engaging STAT1 and STAT3 [16]. Transcriptional regulators, including c-MYC, NF-κB, and epigenetic modulators such as BRD4, can cooperate with STAT signaling to fine-tune ligand expression [17]. Understanding these networks is critical for therapeutic interventions, as modulating upstream regulators may influence the response to immune checkpoint blockade [18].

PD-1, the receptor for PD-L1 and PD-L2, is primarily expressed on activated and exhausted T-cells, and its abundance reflects the functional state of the immune response [19]. Alterations in PD-1 expression in RCC relative to normal kidney tissue may provide insights into immune dysfunction and T-cell exhaustion. PD-L2, although less characterized, similarly mediates inhibitory signaling upon engagement with PD-1 [12]. Combined evaluation of receptors and their ligands may therefore offer a more accurate assessment of immune checkpoint activity, as demonstrated in human melanoma and NSCLC [12]. Paired analyses in RCC are very limited, representing a critical gap in our understanding of checkpoint regulations and informing immunotherapy strategies.

In the present study, we aimed to characterize the regulation of the PD-1 immune checkpoint pathway in ccRCC by integrating analyses of paired human tumorous and adjacent non-tumorous kidney tissues with corresponding in vitro experiments using human ccRCC cell lines. Specifically, we quantified the expression of PD-L1, PD-L2, and PD-1 in patient samples and investigated the time-dependent regulation of PD-L1 and PD-L2 following IFN-γ stimulation in renal carcinoma cell lines, together with the involvement of STAT1 and STAT3 signaling.

By combining paired human tissue data with dynamic in vitro analyses, our recent study can provide a comprehensive and biologically relevant view of PD-1 pathway regulation in ccRCC and also improve our understanding of the immune escape mechanisms and biomarker interpretations related to modern immunotherapy.

## 2. Materials and Methods

### 2.1. Study Population and Tissue Collection

Primary paired tumorous and adjacent non-tumorous renal tissue samples were obtained from 27 patients with histologically confirmed renal cell carcinoma (RCC) who underwent surgical resection at the Department of Urology, University of Debrecen. Tissue specimens were snap-frozen in liquid nitrogen immediately after excision and stored at −80 °C until analysis.

The study was conducted in accordance with the Declaration of Helsinki and was approved by the Medical Ethics Committee of the University of Debrecen (UD REC/IEC 4831-2017). Written informed consent was obtained from all participants prior to sample collection.

Tumors were staged using the TNM staging system of the International Union Against Cancer. Histological grading was performed in accordance with the World Health Organization classification.

### 2.2. Cell Culture and In Vitro Treatments

Human ccRCC cell lines CAKI-2 (HTB-47) and A-498 (HTB-44) were obtained from the American Type Culture Collection (ATCC, Manassas, VA, USA). Cells were maintained in Iscove’s Modified Dulbecco’s Medium (IMDM-Biosera, Cholet, France) supplemented with 10% fetal bovine serum, 100 U/mL penicillin, 100 µg/mL streptomycin, and then cultured at 37 °C in a humidified atmosphere containing 5% CO_2_.

Cells were grown in T75 flasks and passaged two to three times per week. Only low-passage cells (passages ≤ 10) were used for experiments.

### 2.3. Interferon-γ (IFN-γ) Treatment

To investigate the effect of interferon-γ (IFN-γ) on immune checkpoint regulation, CAKI-2 and A-498 cells were treated with recombinant human IFN-γ (BioLegend Way, San Diego, CA, USA) at a final concentration of 10 ng/mL for 2, 6, 12, 24, 48, and 72 h. Untreated cells served as controls.

### 2.4. RNA Extraction and cDNA Synthesis

#### 2.4.1. Total RNA Isolation

Total RNA was isolated from paired human renal tissue samples and cultured renal carcinoma cell lines using TRI Reagent (Molecular Research Center Inc., Cincinnati, OH, USA), according to the manufacturer’s instructions.

Approximately 30 mg of frozen tissue was homogenized in 0.5 mL TRI Reagent using an Ultra-Turrax homogenizer (IKA Labortechnik, Staufen, Germany). Following chloroform extraction and centrifugation, the aqueous phase was collected, and RNA was precipitated with isopropanol, washed with 75% ethanol, and dissolved in nuclease-free water.

For the in vitro experiments, CAKI-2 and A-498 cells were harvested at approximately 80% confluence and lysed directly in TRI Reagent. For IFN-γ-treated samples, RNA was isolated at the indicated time points using the same protocol.

Genomic DNA contamination was eliminated by DNase I treatment (rDNase Set, Macherey–Nagel, Düren, Germany). RNA concentration and purity were determined using a NanoDrop ND-1000 spectrophotometer (Nanodrop Technologies, Wilmington, DE, USA). RNA samples were stored at −70 °C until further use. Parallel cell lysates were prepared for protein analysis. Lysates were prepared from the same samples for subsequent protein analysis.

#### 2.4.2. Reverse Transcription

Complementary DNA (cDNA) was generated from 250 ng of total RNA using the Tetro cDNA Synthesis Kit (Bioline, London, UK) according to the manufacturer’s instructions. Each 20 µL reverse transcription reaction contained 1 µL random hexamer primers, 1 µL 10 mM dNTP mix, 4 µL 5× RT buffer, 1 µL RiboSafe RNase inhibitor, 1 µL Tetro Reverse Transcriptase, and nuclease-free water to a final volume of 20 µL. Reverse transcription was performed on a Bio-Rad C1000 Touch Thermal Cycler (Bio-Rad Laboratories, Hercules, CA, USA) with a 96-well reaction module, using the following program: 25 °C for 10 min, 45 °C for 30 min, and 85 °C for 5 min.

#### 2.4.3. Semi-Quantitative RT-PCR for PD-L1 and PD-L2

Co-expression of PD-L1 and PD-L2 mRNA in human renal tumor tissues was examined by RT-PCR. cDNA was amplified using gene-specific primers for PD-L1, PD-L2, and β-actin (Table 1). PCR reactions were carried out using 1 µL cDNA in a final volume of 25 µL with MyTaq PCR Master Mix (Bioline, London, UK). The amplification protocol consisted of an initial denaturation at 94 °C for 3 min, followed by 45 cycles of 94 °C for 45 s, 60 °C for 30 s, and 72 °C for 90 s, with a final extension at 72 °C for 10 min. For β-actin, 30 amplification cycles were used.

PCR products were separated on 1.5% agarose gels and visualized using GelRed^®^ nucleic acid stain (Biotium, Cambridge, UK), and 50 bp DNA ladder (Bioline, London, UK) was used for the detection of the PCR product.

Template-free and reverse transcriptase-free controls were included to exclude non-specific amplification and genomic DNA contamination, respectively. A pooled cDNA sample was used as a positive control. β-actin amplification yielded a single specific product in all samples, confirming the quality of the cDNA. At mRNA level, PD-L1 and PD-L2 expressions were further verified using end-point PCR on pooled cDNA samples as a positive control.

#### 2.4.4. Quantitative Real-Time PCR (qRT-PCR)

Expression levels of PD-L1, PD-L2, PD-1, STAT1, and STAT3 were quantified by qRT-PCR using iTaq™ Universal SYBR^®^ Green Supermix on a CFX96 Real-Time PCR Detection System (Bio-Rad, Hercules, CA, USA).

Each 20 µL reaction volume contained 10 µL SYBR Green Supermix, 0.5 µL forward and reverse primers (final concentration 250 nM), 2 µL cDNA template and was completed with nuclease-free water. Primer sequences are listed in Table 1.

The amplification program consisted of 95 °C for 10 min, followed by 45 cycles of 95 °C for 15 s and 60 °C for 1 min. Melt-curve analysis was performed to confirm the specificity of amplification.

All reactions were performed in triplicate. β-actin was used as the internal reference gene after validation of its stable expression across experimental conditions. Relative mRNA expression levels were calculated using the 2^−ΔΔCt^ method in tissue and tumorous samples and expressed relative to the corresponding control samples, or as a fold change on a log_2_ scale in the case of cellular experiments.

### 2.5. Protein Extraction and Western Blot Analysis

Protein lysates were prepared from paired tumorous and adjacent non-tumorous renal tissue samples (n = 5 pairs). Protein lysates were also prepared from CAKI-2 and A-498 cells. HPRT was used as a loading control for normalization.

Tissue samples (30–40 mg) and cultured cells were lysed in M-PER buffer (Thermo Fisher Scientific, Waltham, MA, USA) supplemented with protease and phosphatase inhibitors. Protein concentrations were determined using the bicinchoninic acid (BCA) assay.

Equal amounts of protein (40 µg) were mixed with 4× Laemmli buffer, boiled at 95 °C for 8 min, separated on 10% SDS-PAGE gels, and transferred onto PVDF membranes. Membranes were blocked with 5% non-fat milk in TBST (TBS with 0.01% Tween) for 1 h and incubated overnight at 4 °C with primary antibodies against PD-L1, PD-L2, STAT1, STAT3, and HPRT.

After incubation with HRP-conjugated secondary antibodies, signals were detected using enhanced chemiluminescence. Densitometric analysis was performed using Bio-Rad Image Lab software (v5.2.1, Bio-Rad Laboratories, Hercules, CA, USA). Protein expression levels were normalized to HPRT. The antibodies used for Western blotting are listed in Table 2.

### 2.6. Statistical Analysis

Cellular experiments were performed in three independent biological replicates (n = 3), and each biological replicate was measured in triplicate technical replicates for qRT-PCR. Data are presented as mean ± standard error of the mean (± SEM), with *p* < 0.05 considered statistically significant.

Raw data were processed and mean values were calculated using Microsoft Excel (Office Professional Plus 2016, Microsoft, Redmond, WA, USA), and statistical analyses were performed using GraphPad Prism 10.3.1 (GraphPad Software Inc., San Diego, CA, USA).

Pairwise comparisons in tissue-based analyses were performed by Wilcoxon signed-rank test. For cellular qRT-PCR experiments, one-way ANOVA followed by Sidak’s multiple comparison test was used, and the adjusted *p* values were calculated. In normally distributed datasets, Pearson’s correlation coefficient (r) was calculated to assess linear associations, with 95% confidence intervals (CI). The Spearman correlation method was used in not normally distributed datasets. Correlations with r > 0.6 were considered strong.

For Western blot analyses, densitometric values were normalized to the corresponding housekeeping protein and expressed relative to untreated controls. Band intensities were quantified with Image Lab software (version 5.2.1, Bio-Rad Laboratories Inc., Hercules, CA, USA).

## 3. Results

### 3.1. Clinicopathological Characteristics of the Patients

In our study, tumorous and non-tumorous sample pairs from 27 patients were investigated. The clinicopathological data of the patients are shown in Table 3. The majority of the patients were male (23 patients, 85%), whereas four individuals were female (15%), with a total median age of 68 years (range: 53–84). None of the patients had a history of other malignancies or serious infections, and none of them had received radiotherapy, chemotherapy, immunotherapy, or neoadjuvant treatment before surgery. All patients underwent curative surgical resection or laparoscopic nephrectomy. Histopathological evaluation showed that all tumors were ccRCC. According to tumor grade, one case was G1 (3.7%), six were G2 (22.2%), seventeen were G3 (63.0%), and three were G4 (11.1%). Information on smoking status was not available (Table 3).

### 3.2. Semi-Quantitative RT-PCR Analyses for PD-L1 and PD-L2

End-point RT-PCR was performed to investigate the mRNA expression of PD-L1 and PD-L2 in human renal cell carcinoma tissue samples. Template-free and reverse transcriptase-negative controls were included in all reactions to exclude non-specific amplification and genomic DNA contamination. Amplification of the housekeeping gene β-actin yielded a single specific product in all samples, confirming RNA integrity and successful cDNA synthesis.

**Figure 1:**
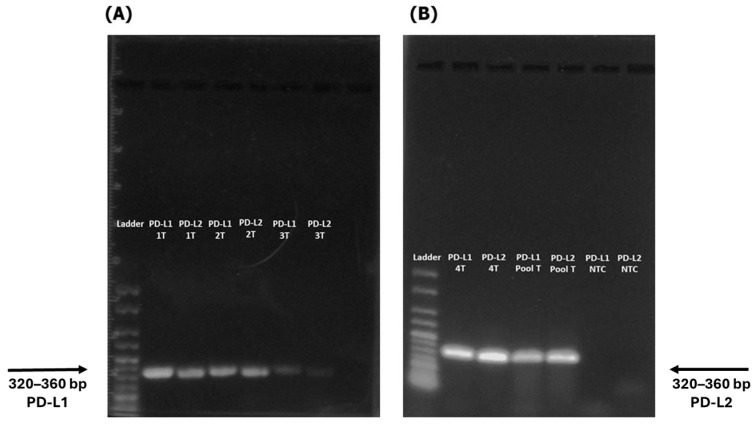
Representative RT-PCR analysis of mRNA for PD-L1 and PD-L2 in 4 human ccRCC specimens. The size of the PCR products was 320–360 bp, as expected for PD-L1 (**A**), and 320–360 bp, as expected for PD-L2 (**B**). Ladder, molecular marker (50-bp DNA ladder). Pool T: positive control for detection of PD-L1. Pool 2: positive control for PD-L2 (pooled cDNAs of the studied tumorous samples). Lanes of NTCs for PD-L1 and PD-L2: no template controls. Samples 1–4 representative tumorous part of the studied human kidney cancer specimens. Bands labeled 1–4 correspond to patient samples 4, 13, 8, and 22, respectively.

Importantly, concurrent expression of PD-L1 and PD-L2 was observed in all tumor specimens. PCR amplification using gene-specific primer pairs yielded single products of the expected sizes for PD-L1 and PD-L2 (approximately 320–360 bp for each target) (Figure 1). Both PD-L1 and PD-L2 transcripts were detected in all analyzed tumor samples, indicating co-expression of the two genes in all specimens. Representative agarose gel images from selected tumor samples illustrate the expression patterns of PD-L1 and PD-L2 (Figure 1).

### 3.3. Analyses of the Expression of mRNA for PD-L1, PD-L2, and PD1 in Human Renal Tissue Samples

The mRNA expression levels of the immune checkpoint ligands PD-L1 and PD-L2, as well as the receptor PD-1, were quantified in 27 paired renal cell carcinoma tissues and matched adjacent non-tumorous renal samples by quantitative real-time PCR.

**Figure 2:**
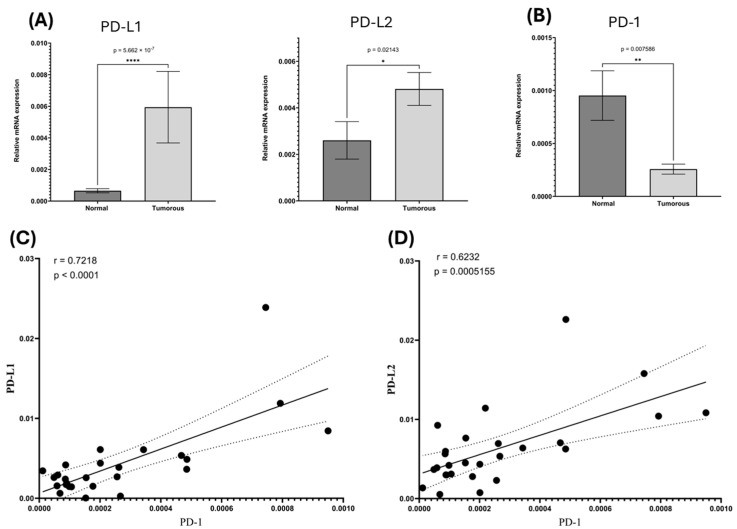
mRNA expression of PD-L1, PD-L2, and PD-1 in renal cell carcinoma samples and adjacent non-tumorous tissues, and correlation between PD-1 and its ligands. For each sample 40 ng of cDNA was used, and β-actin served as the housekeeping gene. Relative mRNA expression levels of PD-L1 and PD-L2 (**A**) in paired RCC samples and matched adjacent non-tumorous renal tissue samples (n = 27), determined by quantitative real-time PCR. (**B**) Relative mRNA expression levels of PD-1 in the same paired tissue set. Differences between tumor and non-tumorous tissues were analyzed using a paired statistical test (*p* = 0.007586); (**C**) Correlation analysis between PD-1 and PD-L1 mRNA expression levels in RCC shows positive correlation (r = 0.7218). (**D**) Correlation analysis between PD-1 and PD-L2 mRNA expression levels in RCC tissues showed strong positive correlation (r = 0.6232). Correlations were assessed using Pearson’s correlation coefficient. In panels C and D, solid lines represent linear regression fits, and shaded areas indicate the 95% confidence intervals. Data are presented as ± standard error of mean (± SEM). The mRNA expression of the paired tissue samples was compared with Wilcoxon signed-rank test (* *p* < 0.05, ** *p* < 0.01, **** *p* < 0.0001).

Expression levels of mRNA for both PD-L1 and PD-L2 were increased significantly in tumor tissues compared with the corresponding non-tumorous renal samples (Figure 2A). In contrast, mRNA expression of PD-1 was significantly lower in RCC tissues relative to the paired adjacent normal tissues (*p* = 0.07586) (Figure 2B).

Correlation analyses performed in tumor samples revealed a significantly positive association between the expression of mRNA for PD-1 and PD-L1 (r = 0.7218, *p* <0.0001; Figure 2C). Additionally, the expression of mRNA for PD-1 showed a significantly positive correlation with PD-L2 mRNA levels (r = 0.6232, *p* = 0.0005155; Figure 2D).

These results indicate that, although PD-1 expression is overall reduced in renal tumor tissues compared with adjacent non-tumorous samples, tumors with higher PD-1 expression also exhibit higher expression of its ligands, PD-L1 and PD-L2. This coordinated expression pattern might support the presence of an actively regulated PD-1 immune checkpoint axis in the renal cancer microenvironment.

**Figure 3:**
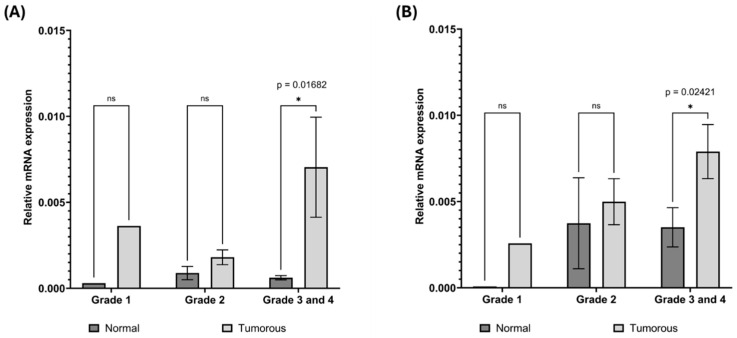
Pathological grade-dependent mRNA expression of PD-L1 (**A**) and PD-L2 (**B**) in renal cell carcinoma. Tumor tissues (light gray) and matched adjacent normal tissues (dark gray) are shown. Expression levels were measured by qRT-PCR (mean ± SEM, triplicates), using 40 ng cDNA per sample and β-actin as housekeeping gene. Statistical analysis: two-way ANOVA with Sidak multiple comparisons. * *p* < 0.05; ns = not significant. (Due to the relatively small cohort size (n = 27) and the uneven distribution of tumor grades (with a predominance of Grade 3 cases), these findings could only be considered exploratory rather than definitive).

To investigate the association among immune checkpoint ligand expressions and pathological grades, PD-L1 and PD-L2 mRNA levels were analyzed in renal tumor tissues and their matched adjacent non-tumorous counterparts (Figure 3A,B).

In tumorous samples the expression of PD-L1 showed a progressive increase with increasing pathological grade, with the highest mean expression observed in Grade 4 tumors (Figure 3A). In contrast, PD-L2 expression in tumor tissues varied across grades and reached its highest level in Grade 3 and 4 samples (Figure 3B).

Across all pathological grades, tumor tissues generally exhibited higher PD-L1 and PD-L2 mRNA expressions compared with the corresponding adjacent normal renal tissues. These findings indicate that the two immune checkpoint ligands display distinct grade-associated expression patterns during renal cancer progression. PD-L1 shows a trend toward enrichment in high-grade tumors, whereas PD-L2 expression peaks at intermediate-to-high pathological grades.

Of course, the relatively small number of tissue samples and the unequal distribution of pathological grades may influence the grade-based expression patterns of PD-L1 and PD-L2 at mRNA levels in the analyzed renal cancer tissues.

### 3.4. Analyses of the Expression of mRNA for STAT1 and STAT3 in Human Renal Cancer Tissues

Expression of mRNA for STAT1 and STAT3 was analyzed in paired human ccRCC specimens and adjacent non-tumorous renal tissue samples by quantitative real-time PCR (qRT-PCR). In addition, the relationship among STAT1/STAT3 expressions and the immune checkpoint ligands PD-L1 and PD-L2 was also examined.

**Figure 4:**
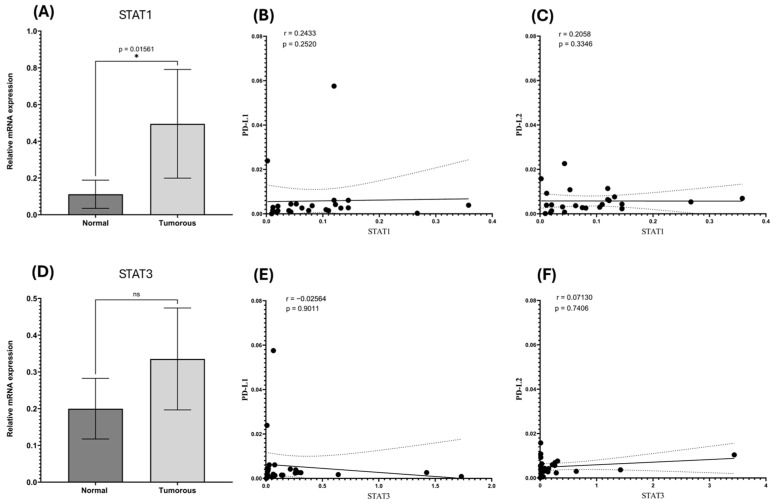
Expression of mRNA for STAT1 and STAT3 and their association with PD-L1 and PD-L2 in human ccRCC tissues. (**A**) Relative STAT1 mRNA expression in paired normal and tumorous ccRCC tissue samples measured by qRT-PCR. STAT1 expression is significantly increased in tumor tissues (*p* = 0.01561). (**B**) Correlation between STAT1 and PD-L1 mRNA expression in tumor samples (r = 0.2433, *p* = 0.2520). (**C**) Correlation between STAT1 and PD-L2 mRNA expression (r = 0.2058, *p* = 0.3346). (**D**) Relative STAT3 mRNA expression in paired normal and tumorous samples. No significant difference is observed between the two groups (ns). Bar graphs show relative expression values normalized to β-actin housekeeping gene and are presented as the ± standard errors of mean (±SEM, log_2_ fold change). (**E**) Correlation between STAT3 and PD-L1 mRNA expression (r = −0.2564, *p* = 0.9011). (**F**) Correlation between STAT3 and PD-L2 mRNA expression (r = 0.07130, *p* = 0.7406). In the scatter plots, each dot represents an individual sample. Solid lines indicate linear regression fits and dashed lines represent the 95% confidence intervals. Statistical significance was defined as *p* < 0.05. Correlation analysis was made by the Spearman method, while mRNA expression of the paired tissue samples was compared with Wilcoxon signed-rank test (* *p* < 0.05).

mRNA expression of STAT1 was significantly higher in tumorous tissues compared with the matched normal tissue counterparts (*p* = 0.01561), indicating transcriptional upregulation of STAT1 in ccRCC (Figure 4A).

In contrast, STAT3 mRNA levels did not differ significantly between tumorous and non-tumorous samples (ns), suggesting the absence of a consistent transcriptional change of STAT3 in ccRCC (Figure 4D).

Correlation analyses revealed no significant association between STAT1 or STAT3 expression and PD-L1 or PD-L2 transcript levels. Specifically, STAT1 expression showed no significant correlation with PD-L1 (r = 0.2433, *p* = 0.2520) or with PD-L2 (r = 0.2058, *p* = 0.3346). Likewise, STAT3 expression was not significantly correlated with PD-L1 (r = −0.2564, *p* = 0.9011) or PD-L2 (r = 0.07130, *p* = 0.7406) (Figure 4).

Taken together, these results indicate that although STAT1 is significantly upregulated at the transcriptional level in ccRCC tissues, neither STAT1 nor STAT3 mRNA expressions show a direct linear relationship with PD-L1 or PD-L2 expression in this cohort. The relatively small number of tissue samples and the unequal distribution of pathological grades may have influenced the expression patterns of PD-L1 and PD-L2 at mRNA levels in the analyzed renal cancer tissues.

### 3.5. Analysis of PD-L1 and PD-L2 Proteins in Representative Human Kidney Cancer Specimens and Their Adjacent Healthy Tissue Samples

**Figure 5:**
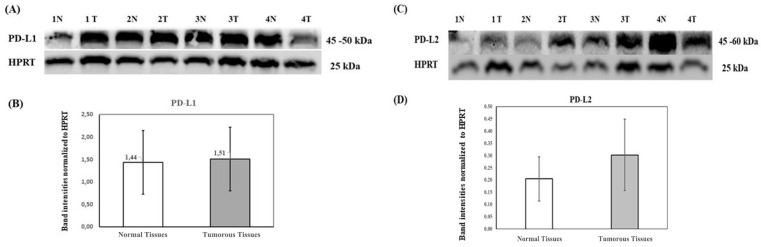
Representative Western blots from 4 paired tissue samples are shown. Individual sample intensities are shown in the (**A**,**C**) and mean ± SEM for normal versus tumor tissues in the bottom of the graph (**B**,**D**). PD-L1 levels are slightly higher in tumor tissues, with sample-to-sample variability. (**C**,**D**) Representative Western blots and quantification of PD-L2 in the same tissue samples. PD-L2 expression shows a similar trend, with modestly increased levels in tumor tissues. Bands labeled 1–4 correspond to patient samples 10, 11, 5, and 3, respectively. For each sample, 40 µg of total protein was separated by SDS-PAGE and transferred to membranes. Detection was performed using monoclonal antibodies specific for PD-L1 (E1L3N^®^) and PD-L2 (D7U8C). Quantification of PD-L1 (**A**) and PD-L2 (**C**) protein in normal (N) and tumorous (T) renal tissue samples (n = 4). Band intensities were quantified with Image Lab software (version 5.2.1, Bio-Rad Laboratories Inc., Hercules, CA, USA). (Due to limited sample size, these data are illustrative and not intended for quantitative comparison. HPRT was used as a loading control based on prior validation of its stable expression in kidney tissues).

The protein expression of PD-L1 and PD-L2 was analyzed by Western blotting in paired human renal tumorous tissues and their adjacent non-tumorous counterparts. PD-L1 and PD-L2 were detected using specific monoclonal antibodies, with HPRT used as a loading control. Among the four representative tissue pairs analyzed, tumorous and adjacent healthy samples showed heterogeneity in the expression of PD-L1 and PD-L2 proteins (Figure 5A–D). Consistent with this variability, quantitative analysis revealed a trend toward increased PD-L1 and PD-L2 expression in tumorous tissues, although substantial inter-sample heterogeneity was observed (Figure 5A–D). This data indicates heterogeneous regulation of PD-L1 and PD-L2 proteins in renal cancer tissues. (As the Western blot analysis was performed only on a limited subset of four paired tissue samples due to restricted tissue availability, these data are intended as representative illustrations rather than quantitative assessment across the full cohort).

### 3.6. Time-Dependent Expression of mRNA for PD-L1 and PD-L2 in A-498 and CAKI-2 RCC Cells After Interferon-γ (IFN-γ) Treatment

To investigate the transcriptional regulation of PD-L1 (CD274) in renal carcinoma cells, CAKI-2 and A-498 cells were treated with 10 ng/mL IFN-γ and the expression of mRNA for PD-L1 was analyzed at multiple time points.

**Figure 6:**
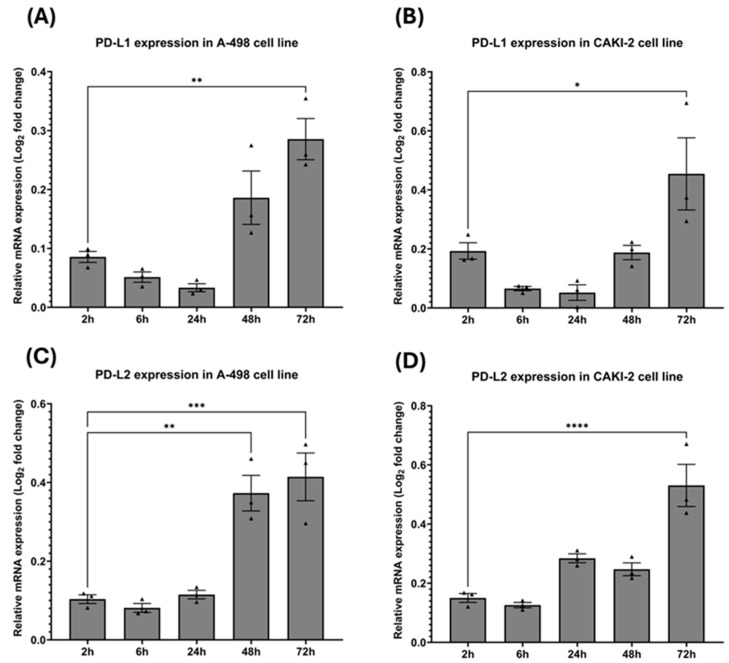
PD-L1 and PD-L2 expressions in A-498 and CAKI-2 cells over time after IFN-γ treatment. (**A**) PD-L1 mRNA expression in A-498 cells at 2 h, 6 h, 24 h, 48 h, and 72 h. Low levels at 2 h and 24 h, significant increase at 48 h and 72 h (*p* = 0.0013). (**B**) PD-L1 mRNA expression in CAKI-2 cells shows a similar temporal increase, significant at 72 h (*p* = 0.0398). (**C**) PD-L2 expression in A-498 cells increases over time, statistically significant difference between 2 h and 48 h (*p* = 0.0011) and the difference is peaking at 72 h (*p* = 0.0004). (**D**) PD-L2 mRNA expression in CAKI-2 cells shows a time-dependent increase, with highest induction at 72 h (*p* < 0.0001). Experiments were carried out in three replicates marked in the figures with triangles. Data represent the mean ± standard error of the mean (± SEM). Relative expression levels of the samples were calculated using the 2^−∆∆CT^ method and normalized to their respective untreated controls (not shown in the figure); represented as log_2_ fold change. One-way ANOVA with Sidak’s multiple comparison test was used to calculate adjusted *p* values to see the significant differences (* *p* < 0.05, ** *p* < 0.01, *** *p* < 0.001, **** *p* < 0.0001).

In the A-498 cell line after IFN-γ treatment, PD-L1 expression increased over time. At 2 h and 24 h, expression levels were low, followed by a significant increase at 48 h and 72 h (*p* = 0.0013), with the highest level observed at 72 h. In CAKI-2 cells, a similar temporal increase in PD-L1 expression was observed after IFN-γ treatment, with a significant rise at 72 h (*p* = 0.0398) (Figure 6A,B)**.**

After IFN-γ treatment, PD-L2 expression also displayed a time-dependent increase. In A-498 cells, significant upregulation occurred between 48 h and 72 h (*p* = 0.0011), peaking at 72 h. In CAKI-2 cells, PD-L2 expression showed an increase over time, with the most significant induction at 72 h (*p* = 0.0004), indicating that this cell line is particularly sensitive to PD-L2 upregulation (Figure 6C,D).

Overall, after IFN-γ treatment, both cell lines exhibited time-dependent upregulation of PD-L1 and PD-L2, with PD-L2 induction being most pronounced in CAKI-2 RCC cells.

### 3.7. Time Dependent Expression of Proteins for PD-L1, PD-L2, STAT1, and STAT3 in A-498 and CAKI-2 ccRCC Cell Lines After Interferon-γ Treatment

IFN-γ signaling activates the JAK–STAT pathway. STAT1 is a key transcriptional regulator of PD-L1 and PD-L2 expression in tumor and immune cells, while STAT3 has been implicated in context-dependent modulation of immune checkpoint regulation. STAT1-mediated induction of PD-L1 following IFN-γ stimulation was previously demonstrated in several tumor models, including renal carcinoma, while STAT3 activity was linked to immune-evasive signaling and checkpoint regulation in cancer cells [20,21].

To investigate the dynamic regulation of immune checkpoint-related proteins and the potential involvement of STAT signaling, CAKI-2 and A-498 renal carcinoma cells were treated with IFN-γ. Protein expressions of PD-L1, PD-L2, STAT1, and STAT3 were analyzed 2, 6, 24, 48, and 72 h after IFN-γ treatment compared to untreated controls by Western blotting.

**Figure 7:**
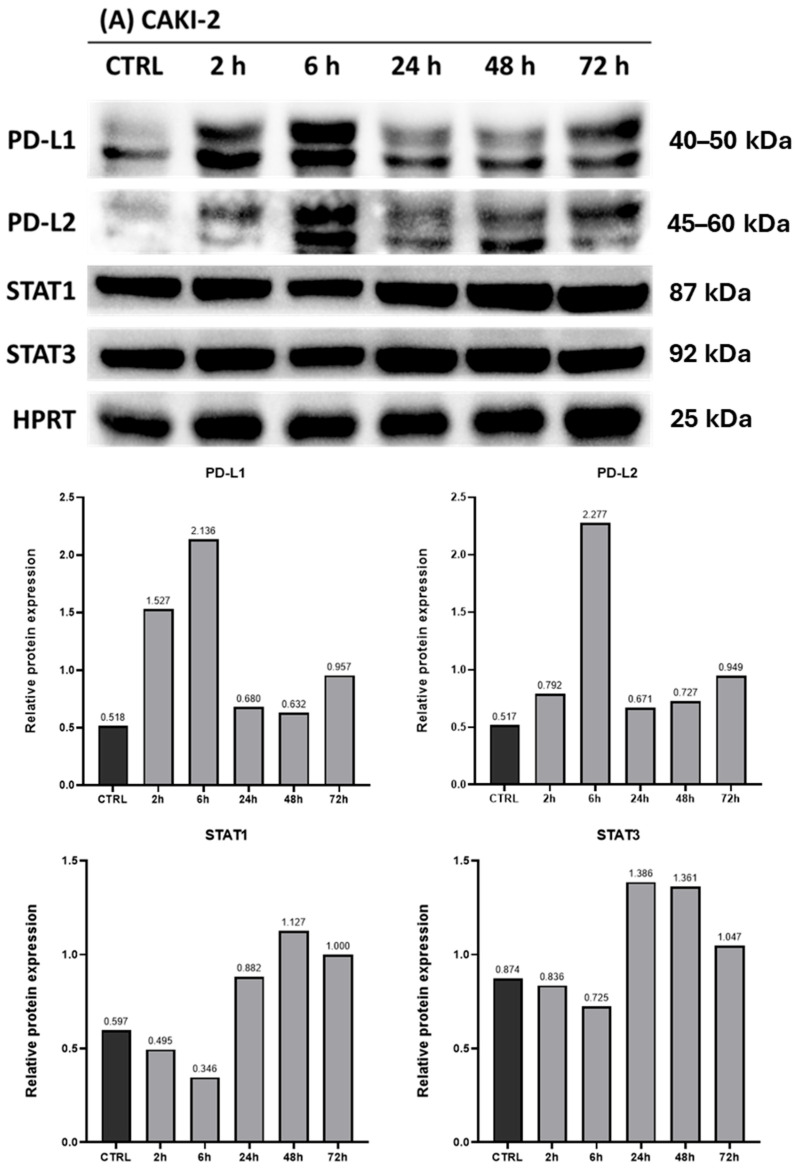

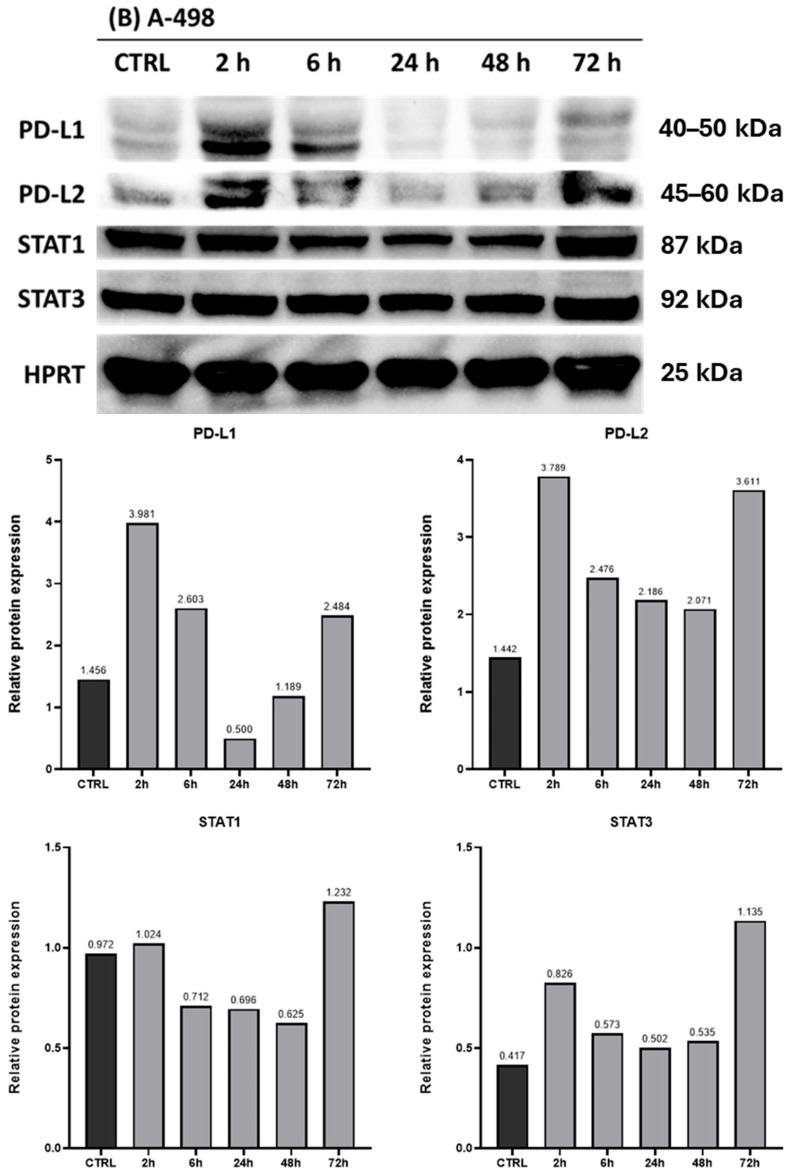
IFN-γ-induced modulation of PD-L1, PD-L2, STAT1, and STAT3 in CAKI-2 and A-498 cells. CAKI-2 and A-498 renal carcinoma cells were treated with IFN-γ and collected at 0, 2, 6, 24, 48, and 72 h. Protein expressions of PD-L1, PD-L2, STAT1, and STAT3 were analyzed by Western blotting, with HPRT as the housekeeping control. Representative blots and quantification are shown in (**A**,**B**). IFN-γ induced time-dependent upregulation of PD-L1 and PD-L2, accompanied by increased STAT1 and STAT3 level. PD-L1 showed pronounced induction at later time points.

CAKI-2 cells exhibited similar trends in PD-L1 and PD-L2 expressions. Both PD-L1 and PD-L2 displayed marked increase 6 h after IFN-γ treatment, followed by a decrease after 24 h. However, protein levels began to increase again 72 h after treatment. STAT1 upregulation was delayed, becoming marked at 48 h, whereas STAT3 protein showed a similar pattern. These results demonstrate that cell line-specific temporal regulation of PD-L1 and PD-L2 in response to IFN-γ treatment might be regulated by STAT1/STAT3 signaling (Figure 7A).

In A-498 cells, IFN-γ induced pronounced, time-dependent changes in PD-L1 and PD-L2 protein levels. PD-L2 was increased after IFN-γ treatment. PD-L1 displayed a biphasic pattern, with early induction at 2–6 h, a relative decrease at 24–48 h, and renewed upregulation at 72 h. STAT1 and STAT3 proteins were somehow upregulated throughout the time course (Figure 7B).

Overall, IFN-γ treatment generated time-dependent and cell line-specific upregulation of PD-L1 and PD-L2, with STAT1 playing a dominant role and STAT3 contributing minimally under these conditions. These results highlight the dynamic regulation of immune checkpoints in renal carcinoma cells and provide novel insight into IFN-γ-mediated signaling.

Notably, the temporal changes observed at protein levels did not fully reflect the mRNA expression profiles detected by qRT-PCR for either PD-L1 or PD-L2. Especially at later time points following IFN-γ stimulation. Both PD-L1 and PD-L2 mRNA levels showed similar delayed transcriptional responses to IFN-γ treatment. Protein expression of both ligands, however, displayed an early increase accompanied by STAT1 induction, with the most pronounced accumulation observed at 2–6 h. These findings indicate a time and cell type dependent and multi-layered regulation of PD-1 ligands, suggesting that protein-level assessment may more accurately reflect the functional immune checkpoint status under prolonged inflammatory stimulation [22,23].

## 4. Discussion

Programmed cell death protein-1 (PD-1) and its ligands PD-L1 and PD-L2 constitute a central immune-checkpoint pathway that regulates T-cell activation and peripheral tolerance. Engagement of PD-1 by PD-L1 or PD-L2 expressed on tumor or immune cells suppresses effector T-cell function, thereby facilitating immune escape and promoting tumor progression. Blockade of the PD-1/PD-L1 axis has become a potential therapeutic strategy in several malignancies, including RCC, underscoring the clinical and biological importance of this pathway [24].

RCC is characterized by a highly heterogeneous and immunologically active TME composed of tumor cells, infiltrating lymphocytes, macrophages, stromal cells, cytokines, and extracellular matrix components [25]. This complex and heterogeneous milieu profoundly influences tumor progression, immune-checkpoint expression, and therapeutic response [25,26]. Tumor-infiltrating lymphocytes, regulatory T cells, myeloid-derived suppressor cells, and macrophages collectively contribute to marked inter-tumoral variability in PD-1 ligand expression [25,26]. Clear cell renal cell carcinoma, in particular, represents an immunologically and histologically diverse entity, with structural heterogeneity of the TME being a major determinant of cancer progression and treatment response. It has already been revealed that the structural heterogeneity of TME in ccRCC impacts the clinical outcome and personalized treatment [27]. Some studies have shown that increased expression of PD-L1 and PD-L2 correlates with unfavorable outcome of ccRCC [28,29,30]. Therefore, comprehensive characterization of PD-1 pathway regulation in RCC is essential for improving the understanding of immune-escape mechanisms and for refining biomarker interpretation related to immunotherapy.

In our study, quantitative mRNA analyses of 27 paired ccRCC and adjacent non-tumorous kidney samples demonstrated significant upregulation of PD-L1 and PD-L2 in tumors. These findings are consistent with previous reports linking high PD-1 ligand expression to an immunosuppressive TME [31]. In contrast, PD-1 transcripts were predominantly downregulated in tumor samples, relative to matched normal kidney tissue. Despite lower PD-1 expression, correlation analyses revealed significant positive associations between PD-1 and PD-L1/PD-L2, reflecting PD-1/PD-L1 axis activity in RCC [32].

Across all grades, tumor tissues exhibited higher PD-L1 and PD-L2 expressions than matched normal tissues, although the small sample size and unequal grade distribution limited the statistical power. Therefore, this observation is exploratory due to the limited sample size and uneven grade distribution. However, investigation of PD-L1 and PD-L2 expression in the studied samples by tumor grades revealed distinct expression patterns of the two ligands investigated. Expression of both ligands, PD-L1 and PD-L2, increased progressively in tumorous samples with advancing grades, mainly in Grade 3 and Grade 4 tumors. Several clinical studies and meta-analyses have already shown that PD-L1 expression correlates with adverse clinicopathological features, including higher nuclear grade, larger tumor size, advanced TNM stage, increased risk of lymph-node involvement, and distant metastasis. However, due to the uneven distribution of tumor grades, with a predominance of Grade 3 tumors, which limits the statistical power for grade-stratified analyses, the observed trends in PD-L1/PD-L2 expression across tumor grades should be considered exploratory and require validation in larger, more balanced cohorts. Our results also support the notion that PD-L1 is not only an immune biomarker but also reflects underlying tumor biology linked to disease progression. It may provide complementary prognostic information for proper clinical decisions in RCC [33]. The clinical relevance of PD-L2 appears to be more TME-dependent [32,34].

Western blot analyses of human renal tissue pairs revealed generally higher PD-L1 and PD-L2 protein levels in cancer samples, consistent with transcriptional data. Notably, some specimens displayed lower ligand expression in tumor samples compared with matched normal tissue, reflecting substantial inter-sample heterogeneity. These observations underscore the complexity of immune-checkpoint regulation and the impact of tumor-specific microenvironmental factors [35]. The small number of tissue samples analyzed by Western blot restricts final protein-level conclusions and mainly has an illustrative role in our study.

IFN-γ stimulation of CAKI-2 and A-498 RCC cells induced pronounced, time-dependent changes in PD-L1 and PD-L2 expressions at both mRNA and protein levels. Transcriptional induction was delayed, whereas maximal protein accumulation occurred rapidly, peaking at 2–6 h. Specifically, PD-L1 mRNA expression dropped at 24 h and started to increase after 48 h. Notably, PD-L2 displayed a biphasic pattern, suggesting tighter regulatory control compared with PD-L1 [36]. These temporal differences suggest the role of post-transcriptional and translational regulations in interpreting ligand expression, consistent with observations in other tumor models [22,23].

IFN-γ signaling is primarily mediated through the JAK–STAT pathway. STAT1, a canonical mediator of interferon responses, promotes PD-L1 and PD-L2 transcription, whereas STAT3 is more frequently associated with oncogenic signaling and immune suppression [23,37]. In IFN-γ-treated RCC cell lines, STAT1 and STAT3 proteins levels increased dynamically. However, STAT1 and STAT 3 induction kinetics differed between CAKI-2 and A-498 cells, indicating cell line-specific responses. In patient samples, STAT1 mRNA expression was significantly higher (*p* = 0.01561) in tumor tissues than in normal kidney, whereas STAT3 showed no overall difference but marked inter-patient variability. These findings might indicate that STAT1-driven signaling is a major regulatory axis controlling PD-1 ligand induction in RCC, whereas STAT3 contributes in a context-dependent manner [23,37,38,39]. Although no overall correlation between STAT1 and PD-L1/PD-L2 expression was observed across all samples, this may be explained by tumor heterogeneity and the relatively small sample size. Given that PD-L1 and PD-L2 expression is elevated in higher tumor grades, a positive association with STAT1 becomes detectable in these subsets.

**Figure 8:**
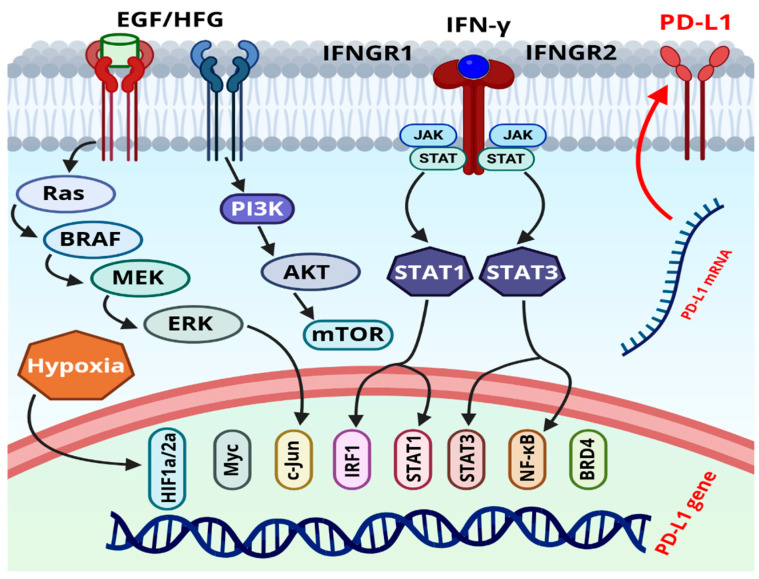
Schematic overview of signaling pathways involved in the regulation of PD-1 ligands in clear cell renal cell carcinoma. Interferon-γ (IFN-γ) binding to the IFNGR1/IFNGR2 receptor complex activates the JAK–STAT pathway, leading to phosphorylation and nuclear translocation of STAT1 and STAT3 and subsequent transcriptional activation of the PD-L1 gene through downstream mediators such as IRF1. Additionally, the diagram summarizes the potential crosstalk between immune-mediated and tumor-intrinsic signaling pathways controlling PD-L1 expression in ccRCC [23,37,38,39].

To provide an integrated interpretation of our experimental findings, a putative schematic overview of the major signaling pathways regulating PD-1 ligands is presented in Figure 8.

Our in vitro data indicate that IFN-γ-mediated activation of the IFNGR1/IFNGR2–JAK–STAT1 axis could be the predominant mechanism driving PD-L1 and PD-L2 induction in RCC cells [23]. Consistent STAT1-associated expression patterns across RCC cell lines support the relevance of this pathway in IFN-γ-mediated immune regulation, although functional validation is still required in future studies.

In parallel, constitutive HIF signaling caused by VHL alterations in ccRCC may contribute to the baseline expression of both PD-L1 and PD-L2 [40,41,42]. According to previous studies, additional tumor-intrinsic signaling pathways, including PI3K–AKT–mTOR and Ras–RAF–MEK–ERK cascades, may also participate in the regulation of PD-L1 and PD-L2 expressions [43]. However, activation of these pathways in response to IFN-γ is highly cell-type-dependent and is not consistently observed. Therefore, the potential involvement of these pathways was not addressed in the present study [44].

PD-1 is primarily expressed by infiltrating immune cells, and the reduced PD-1 transcript levels detected in tumor samples are therefore likely attributable to altered immune-cell composition within the tumor microenvironment [45,46]. The positive intratumoral correlation between PD-1 and PD-L1 expressions in the studied samples further indicates that ligand induction in tumor cells remains closely linked to the presence and activation status of PD-1-expressing immune populations [45,46]. A key limitation of this study is the absence of loss-of-function approaches (e.g., CRISPR/Cas9-mediated inhibition or siRNA-mediated knockdown of STAT1 and STAT3), which precludes definitive conclusions regarding the relative contribution of STAT1 and STAT3 to PD-L1/PD-L2 induction. Future studies using targeted functional inhibition of STAT1 and STAT3 will be necessary to further clarify their specific regulatory roles in PD-L1/PD-L2 expression in ccRCC. In addition, tissue-based methods such as immunohistochemistry or immunofluorescence could provide further validation at the tissue level.

More broadly, cytokine-mediated activation of the JAK–STAT signaling cascade is a central regulatory axis in TME, influencing proliferation, survival, immune surveillance, and immunomodulation [47]. Among STAT family members, STAT1 and STAT3 exert distinct and often opposing functions in tumorigenesis. Persistent STAT3 activation promotes tumor progression and immunosuppression, whereas STAT1 is linked to antitumor immunity [21,47]. PD-L1 expression has been directly associated with both STAT1 and STAT3 activity, indicating convergence of multiple regulatory pathways [48]. According to the literature, STAT1 is the main mediator of IFN-γ signaling and directly drives PD-L1 and PD-L2 expression. In contrast, STAT3 is more often linked to tumor-promoting and immunosuppressive processes in the tumor microenvironment. This may explain why STAT1 shows a strong and consistent increase after IFN-γ stimulation in vitro, while STAT3 expression in patient tissues is more variable and does not show a clear overall increase [48].

Although much of the previous findings for STAT-mediated checkpoint regulation derives from other tumor models, transcriptomic analyses suggest that STAT family members are differentially expressed in RCC. Their expression correlates with immune-cell infiltration and clinical outcome, supporting a functional role of STAT signaling in disease progression [21,49]. Consequently, the balance between STAT1 and STAT3 activity within TME may influence not only tumor progression and immune phenotype but also responsiveness to PD-1/PD-L1-targeted therapies [49]. Our findings indicate that reduced PD-1 expression in tumor tissues likely reflects differences in immune-cell composition which is consistent with previous reports [50,51]. Biologically, PD-L1 and PD-L2 upregulation primarily appears to be mainly associated with IFN-γ-induced STAT1 activation and may reflect an adaptive immune-resistance response within the ccRCC microenvironment. Clinically, this suggests that PD-1 ligand expression may reflect an ongoing but functionally constrained antitumor immune response rather than constitutive immune evasion alone. Consequently, integrated assessment of PD-L1/PD-L2 expression along with indicators of IFN-γ/STAT1/STAT3 pathway activity could help refine patient stratification for immune-checkpoint therapies, although validation in larger, well-characterized cohorts is needed.

The heterogeneity of PD-L1/PD-L2 expressions and STAT signaling observed in our study and in previous reports has important therapeutic implications as well. Tumors with high PD-L1/PD-L2 expressions and active STAT1 signaling may respond better to PD-1/PD-L1 blockade, whereas STAT3-dominant tumors may require more complex combination strategies [43]. Thus, integrated profiling of the expression of these ligands and STAT signaling activity could improve personalized immunotherapy [21,49,52]. Although IFN-γ/STAT1-mediated PD-L1 regulation has been widely described and reported, our study provides ccRCC-specific insights by combining paired human tissue analyses with time-resolved in vitro experiments. We also examined PD-L2, which is less characterized in ccRCC, and observed marked inter-tumoral heterogeneity. Importantly, the lack of direct correlation between STAT1/STAT3 and PD-L1/PD-L2 in patient tissues highlights the complexity of immune-checkpoint regulation in the tumor microenvironment.

In summary, our investigation demonstrates that PD-L1 and PD-L2 are frequently upregulated in ccRCC, likely exhibit grade-specific patterns, and are dynamically regulated by IFN-γ/STAT1 signaling, while PD-1 is more prominent in normal kidney tissue. The pronounced inter-tumoral and temporal heterogeneity highlights the complexity of immune-checkpoint regulation in RCC and underscores the need for integrated profiling to optimize therapeutic strategies [53].

This study has some limitations. We were able to collect and investigate ccRCC samples from 27 patients, analyzing altogether 54 human tissue samples. A major limitation of this study is the uneven distribution of tumor grades, with a predominance of Grade 3 tumors, which limits the statistical power for grade-stratified analyses. Therefore, the observed trends in PD-L1/PD-L2 expression across tumor grades should be considered exploratory and require validation in larger, more balanced cohorts. It is important to note that the relatively small sample size may restrict statistical power, particularly for subgroup analyses. However, the use of paired tumorous and adjacent non-tumorous tissues reduces inter-individual variability and enhances analytical sensitivity; nevertheless, validation in larger, independent cohorts is warranted. In addition, PD-1, PD-L1, and PD-L2 expressions were assessed mainly at the bulk tissue level, precluding precise discrimination between tumor-cell- and immune-cell-derived signals. Future studies using cell-specific methods, such as flow cytometry or immunostaining, are needed to identify which cells express PD-1, PD-L1, and PD-L2. Some of our conclusions are primarily based on two RCC cell lines and IFN-γ stimulation and therefore may not fully reflect the diversity of regulatory pathways operating in vivo. Future studies incorporating larger, clinically annotated cohorts and single-cell or spatial profiling approaches will be required to further validate and extend these findings.

## 5. Conclusions

PD-L1 and PD-L2 are frequently upregulated in ccRCC and are dynamically induced by IFN-γ in association with STAT1 signaling, while STAT3 plays a more limited role. Reduced PD-1 levels may be partly related to differences in immune-cell composition within the tumor microenvironment; however, this interpretation remains indirect due to the use of bulk tissue analysis and requires further validation using single-cell approaches. Our findings highlight the complexity of immune-checkpoint regulation in ccRCC and underscore the value of integrated transcriptional and protein-level profiling for improved biomarker interpretation and immunotherapy guidance. However, due to the lack of loss-of-function validation, the relative contribution of STAT1 versus STAT3 in PD-L1/PD-L2 induction cannot be definitively established, particularly whether PD-L1/PD-L2 induction is primarily driven by STAT1 rather than STAT3.

## Figures and Tables

**Table 1:** Primer sequences used in qRT-PCR analysis.

Gene	Forward Primer (5′ → 3′)	Reverse Primer (5′ → 3′)
PD-L1 (CD274)	TGCCGACTACAAGCGAATTACTG	CTGCTTGTCCAGATGACTTCGG
PD-L2 (PDCD1LG2)	CTCGTTCCACATACCTCAAGTCC	CTGGAACCTTTAGGATGTGAGTG
PD-1 (PDCD1/CD279)	AAGGCGCAGATCAAAGAGAGCC	CAACCACCAGGGTTTGGAACTG
STAT 1	CAC CAGAGCCAATGGAACTT	ACAGAGCCCACTATCCGAGA
STAT 3	CTTTGAGACCGAGGTGTATCACC	GGTCAGCATGTTGTACCACAGG

**Table 2:** List of the antibodies used for Western blots.

Antibody	Origin, Catalog Number	Dilution Applied
PD-L1 (E1L3N^®^) XP^®^ Rabbit mAb	Cell Signaling, #13684	1:1000
PD-L2 (D7U8C) XP^®^ Rabbit mAb	Cell Signaling, #82723	1:1000
Anti-HPRT1 (P00492) Rabbit Mab	BOSTER Biological Technology #M00668	1:1000
STAT 1 Polyclonal Antibody	Elabscience, E-AB-32977	1:1000
STAT 3 Polyclonal Antibody	Elabscience, E-AB-93190	1:1000

**Table 3:** Clinicopathological data of ccRCC patients involved in this study.

	Gender	Age	Histological Type	Grade	TNM	Tumor Size
1	Male	53	ccRCC	G1	pT1a pN0	2.5 cm
2	Male	59	ccRCC	G3	pT1b pN0	6.0 cm
3	Male	71	ccRCC	G3	pT2b pN0	11.0 cm
4	Male	65	ccRCC	G3	pT1b pN0	4.5 cm
5	Male	76	ccRCC	G3	pT1b pN0	5.0 cm
6	Male	56	ccRCC	G2	pT1b pN0	4.5 cm
7	Male	70	ccRCC	G4	T3b pN0pM1	10.0 cm
8	Male	67	ccRCC	G3	pT3a pN0	7.7 cm
9	Male	84	ccRCC	G3	pT1a pN0	2.3 cm
10	Male	80	ccRCC	G4	pT3a pN0	8.7 cm
11	Female	69	ccRCC	G2	pT1b pN0	6.0 cm
12	Female	70	ccRCC	G3	pT1b pN0	6.0 cm
13	Male	74	ccRCC	G3	pT1a pN0	3.5 cm
14	Male	77	ccRCC	G4	pT3a pN1	9.0 cm
15	Male	76	ccRCC	G2	pT1b pN0	5.0 cm
16	Female	68	ccRCC	G2	pT1a pN0	2.8 cm
17	Female	67	ccRCC	G2	pT1b pN0	5.6 cm
18	Male	58	ccRCC	G3	pT1b pN0	6.0 cm
19	Male	69	ccRCC	G3	pT2b pN0	11.0 cm
20	Male	71	ccRCC	G2	pT2b pN0	5.7 cm
21	Male	77	ccRCC	G3	pT3a pN1	6.0 cm
22	Male	71	ccRCC	G3	pT3a pN1	6.0 cm
23	Male	78	ccRCC	G3	pT3a pN1	6.0cm
24	Male	63	ccRCC	G3	pT3a pN1	5.0 cm
25	Male	68	ccRCC	G3	pT3a pN1	8.0 cm
26	Female	71	ccRCC	G3	pT3a pN1	7.0 cm
27	Male	52	ccRCC	G3	pT3a pN1	12.0 cm

Note: pT1a indicates an organ-confined tumor smaller than 4 cm, pT1b refers to an organ-confined tumor measuring 4–7 cm, and pT3a describes tumor invasion into the renal vein or its segmental branches. ccRCC denotes clear cell renal cell carcinoma. N0 indicates absence of lymph-node involvement and distant metastasis, pM1 indicates the presence of distant metastases, and pN1 refers to micrometastases or metastases in regional lymph nodes.

## Data Availability

The data presented in this study are available on request from the corresponding author.

## Abbreviations

The following abbreviations are used in this manuscript:

PD-1	Programmed cell death protein-1
PD-L1	Programmed cell death ligand 1
PD-L2	Programmed cell death ligand 2
STAT1	Signal transducer and activator of transcription 1
STAT3	Signal transducer and activator of transcription 3
ccRCC	Clear cell renal cell carcinoma
RCC	Renal cell carcinoma
TME	Tumor microenvironment
ICI	Immune checkpoint inhibitor
NSCLC	Non-small cell lung cancer
IFN-γ	Interferon-γ
SEM	Standard error of the mean
qRT-PCR	Quantitative Reverse Transcription Polymerase Chain Reaction
IMDM	Iscove’s Modified Dulbecco’s Medium
PVDF	Polyvinylidene difluoride
CTLA-4	Cytotoxic T-Lymphocyte-Associated Protein 4
SDS-PAGE	Sodium dodecyl sulfate–polyacrylamide gel electrophoresis
IFNGR1	Interferon Gamma Receptor 1
IFNGR2	Interferon Gamma Receptor 1
JAK	Janus kinase
PI3K	Phosphoinositide 3-kinase
AKT	Protein Kinase B
mTOR	Mammalian target of rapamycin
